# Isolated bilateral hypoglossal nerve paralysis following an atlanto-occipital dislocation: A case report

**DOI:** 10.3389/fneur.2022.965717

**Published:** 2022-09-14

**Authors:** Tomoo Mano, Saori Tatsumi, Shigekazu Fujimura, Naoki Hotta, Akira Kido

**Affiliations:** ^1^Department of Neurology, Nara Medical University, Kashihara, Japan; ^2^Department of Rehabilitation Medicine, Nara Medical University, Kashihara, Japan; ^3^Medical Technology Center Division, Nara Medical University Hospital, Kashihara, Japan

**Keywords:** bilateral hypoglossal paralysis, dysarthria, dysphagia, vertical atlantoaxial dislocation, acute epidural hematoma

## Abstract

The present report highlights a case of successful treatment of an 11-year-old male patient who presented with an atlanto-occipital dislocation and multiple fractures of the forearm, pelvis, and lower leg because of a fall. The patient experienced dysarthria and paralysis of the tongue, which became completely immobile and could not be moved from side to side, impeding speech. The patient also experienced dysphagia due to the inability to propel food toward the pharynx and chewing attempts resulted in scattering of food residue throughout the oral cavity. The lack of tongue mobility led to saliva accumulation, forcing the patient to swallow frequently, which was possible as larynx movement was unaffected. The other cranial and motor sensory nerves appeared normal. Our diagnostic examinations confirmed the presence of isolated bilateral paralysis of the hypoglossal nerve secondary to traction at the base of the skull. The patient was still unable to protrude his tongue and tongue gradually atrophied two weeks after admission. Electromyography revealed denervation of the tongue and minimal active contraction of the single motor units. Immobilization therapy and rehabilitation therapy were initiated to improve tongue movement, but this was unsuccessful and one month after the accident, the patient's tongue was still atrophied. The patient was placed on a soft food diet and experienced no difficulty in swallowing either saliva or food three months after admission. Tongue mobility was deemed normal. Electromyography six months after the initial episode revealed normal motor unit potentials during contractions. We postulate that compression and stretching of the bilateral hypoglossal nerves against the greater horn of the hyoid bone was a probable cause of the hypoglossal palsy. The use of immobilization and rehabilitation therapy likely supported the recovery of functionality and resulted in a good prognosis.

## Introduction

Isolated hypoglossal nerve palsy is a rare occurrence, commonly associated with other cranial nerve palsies and long tract signs. Hypoglossal nerve palsy is usually unilateral and is associated with the involvement of other cranial nerves and neurological structures (1, 2). Numerous cases of isolated bilateral hypoglossal nerve palsy have previously been reported (3).

In the present case, the isolated bilateral hypoglossal nerve palsy presented as dysarthria, saliva accumulation that forced the patient to swallow frequently, dysphagia in the form of inability to propel food toward the pharynx, and dyspnea in the supine position due to difficulty in raising the tongue.

We describe the case of a patient with isolated bilateral hypoglossal nerve paralysis following traction after an atlanto-occipital dislocation, whose condition improved with cervical spine immobilization and rehabilitation treatment. To our knowledge, bilateral hypoglossal nerve palsy following an atlanto-occipital dislocation has not been previously reported.

## Case description

An 11-year-old boy experienced an accident after falling from the 8th floor of a building while looking down from a balcony. When the emergency medical team arrived, the patient's initial consciousness,using the Glasgow coma scale (eye, verbal and motor (EVM) responses was assessed and found as E3V4M6. A hard neck collar was placed on the patient and he was transferred to our institution. Tracheal intubation was performed because the patient's consciousness level decreased to E2V2M5 and the patient had no focal neurologic deficits during transportation. On arrival, the patient was hemodynamically unstable and ventilator management was initiated.

**Figure 1:**
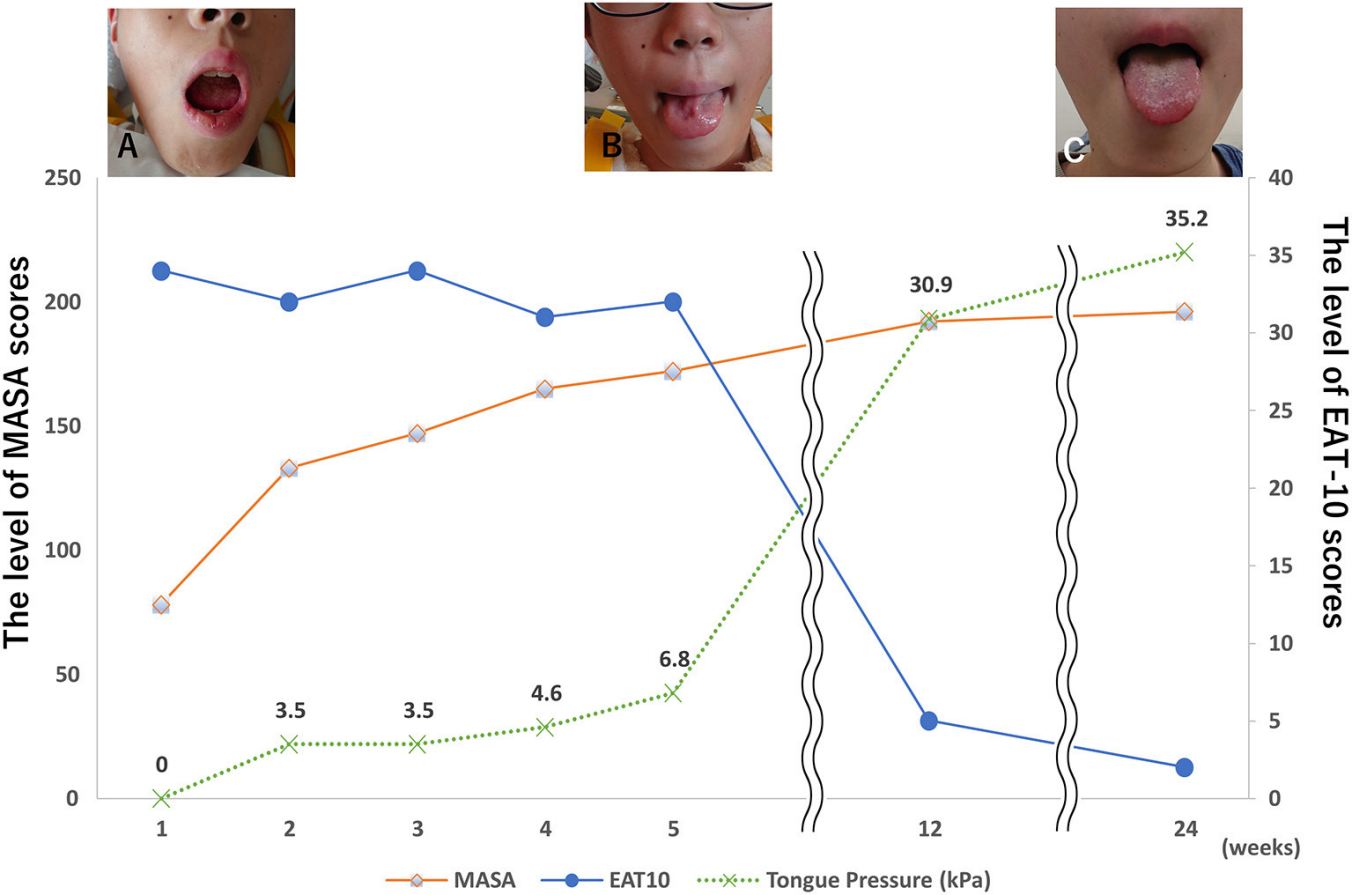
Image inspection. **(A)** A cervical X-ray shows atlanto-occipital dislocation, (a) space available for the cord is 23.02 mm, (b) atlanto-dental interval is 4.64 mm. **(B)** A cervical computed tomography scan shows an acute epidural hematoma in front of the medulla oblongata (red arrow). **(C)** An arterio-venous shunt runs from the dorsal side of the clivus to the sublingual branch (the neuro-meningeal branch of the left ascending pharyngeal artery) to the right cavernous sinus.

The patient underwent X-ray and whole body computed tomography (CT) examinations. Cervical X-ray revealed an atlanto-dental interval of 4.64 mm, space available for the cord at 23.02 mm, a Ranawat value of 16.29 mm, and a Redlund-Johnell value of 46.10 mm. An atlanto-occipital dislocation was diagnosed in the patient (Figure 1A). Immediately after diagnosis, the patient underwent skull traction using halo immobilization to treat the vertical dislocation of the cervical spine. Head and cervical CT revealed an epidural hematoma of the medulla oblongata and vertical dislocation of the cervical spine (Figure 1B). At first, we suspected that the epidural hematoma following the atlanto-occipital dislocation may have caused the bilateral nerve compression. However, this was ruled out, as no other neurological deficits associated with compression by the small epidural hematoma were observed. As the patient was a minor, we had to obtain parental consent for further procedures. Thereafter, we performed angiographic evaluation and selected endovascular therapy to treat the growing hematoma. Transcatheter arterial embolization was performed using a coil to treat an arterio-venous shunt that ran from the dorsal side of the clivus to the sublingual branch, which is the neuro-meningeal branch of the left ascending pharyngeal artery, to the right cavernous sinus (Figure 1C). Whole body CT revealed a pulmonary contusion, fractures in the right skull, right ulna, distal ends of the right and left tibia, and the right talus, and injury to the right pelvic ring. The patient was transferred to the intensive care unit, initiated to sedate with anesthetic, and had no significant period of cough or nausea prior to the first extubation attempt. Physical therapy was initiated two days after admission. We performed a range of motion and respiratory physiotherapy with the patient on the bed. The endotracheal tube was removed three days after admission. Follow-up CT revealed no increase in size of the epidural hematoma.

**Figure 2:**
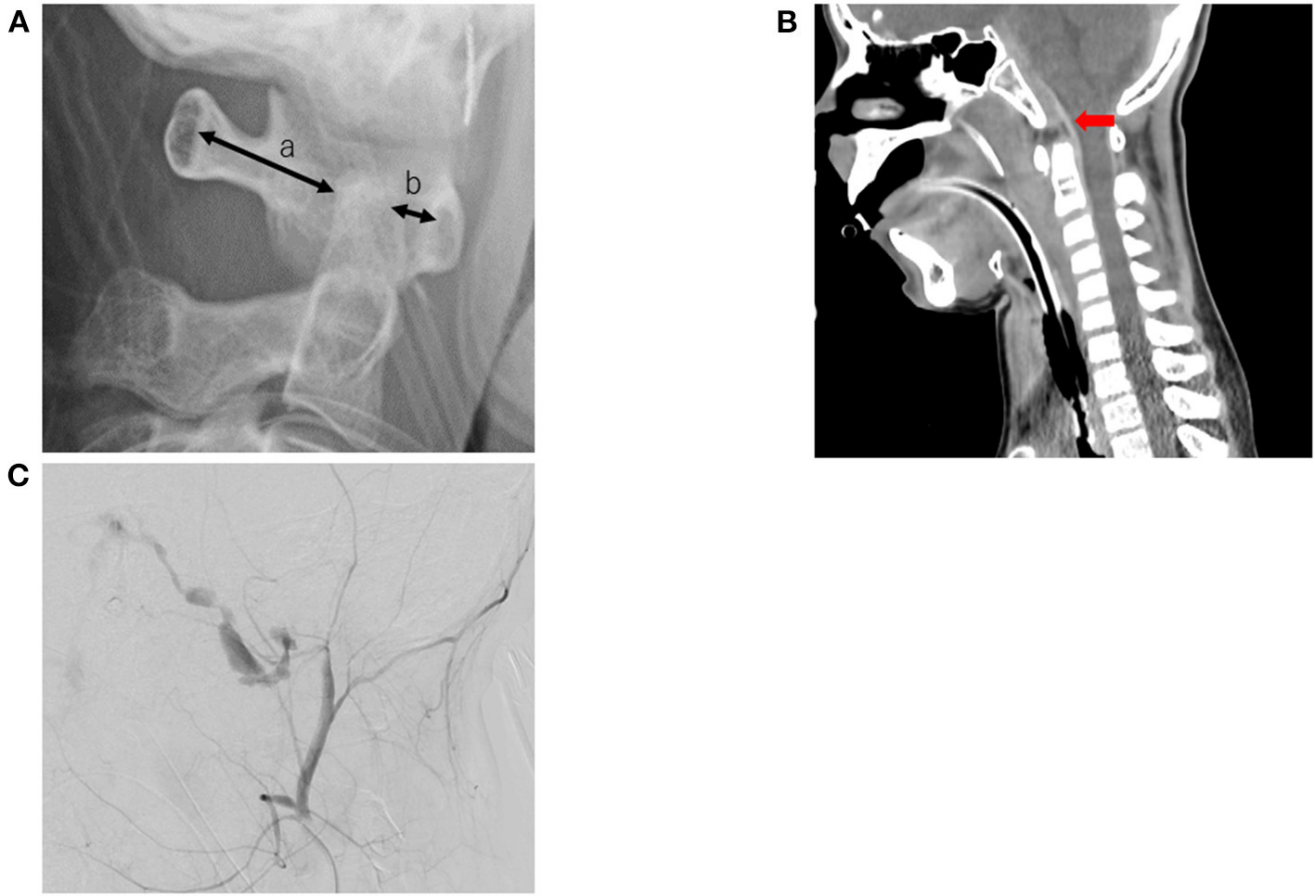
Clinical course. **(A)** The patient's tongue was completely immobile and he was unable to move it from side to side 3 days after the accident. **(B)** The patient's tongue could be moved but was slightly atrophied. **(C)** The patient's tongue could move in full range, and the atrophy improved.

Neurological assessments were performed three days after admission and revealed slurred speech and pronounced dysarthria. The tongue showed diffuse atrophy, fasciculation, and inability to move or protrude the tongue on the floor of the mouth. The patient had decreased intelligibility of vocal sounds related to the tongue but no nasal sounds to his voice. Saliva accumulated in the mouth forcing him to swallow frequently. A detailed neurological assessment revealed no apparent evidence of other cranial nerve involvement. Neither abnormal tendon reflexes nor pathological reflexes, such as Babinski or Chaddoch, were observed. Therefore, we considered the diagnosis of isolated bilateral hypoglossal nerve palsy. A Speech Intelligibility Rating (SIR) test was administered, and the patient's rating was category 4 (4). A clinical evaluation, including Eating Assessment Tool-10 (5), Mann Assessment of Swallowing Ability (MASA) (6), and tongue pressure test (7), was performed (Figures 2A–C).

Speech therapy was initiated four days after admission, consisting of swallowing and vocalization training for 40 min each day. In addition, physical therapy included muscle strengthening and training to get out of bed.

A nasogastric feeding tube was inserted, and swallowing rehabilitation was initiated four days after admission. At 14 days post admission, the patient was still unable to protrude his tongue and the tongue had gradually atrophied. Electromyography revealed signs of neurological denervation of the tongue, such as fibrillation potentials, positive sharp waves, and minimal active contraction of the single motor units. The patient's hypoglossal palsy slowly resolved, and the patient could extend his tongue symmetrically one month after the initial accident. The tongue was slightly atrophied, but the patient's SIR improved to category 2. The patient received a soft food diet and did not experience difficulty in swallowing either saliva or food three months after the initial accident. Tongue mobility was deemed normal, and the SIR improved to category 1. Electromyography six months after the accident revealed normal spontaneous activity, normal motor unit potentials, and no reduced interference patterns.

## Discussion

The hypoglossal nerve innervates the muscles that control tongue movement, except for the palatoglossus muscle, which is vagally innervated and aids in tongue movement necessary for mastication, swallowing, and speech production (8). Hypoglossal nerve palsy rarely occurs in isolation. A previous summary of 100 cases of 12th cranial nerve palsy reported that half of the cases resulted from tumors, of which half were malignant. Other causes included trauma, stroke, neuropathy, surgery, and infection (1). There was no clear mechanism for the hypoglossal nerve injury in this patient. No other cranial nerves were affected, and brain CT revealed normal.

Patients with unilateral hypoglossal nerve palsy may present mild dysphagia or dysarthria; however, most patients have no subjective complaints at all. Typical findings of unilateral nuclear and infranuclear hypoglossal nerve palsy are fasciculation, atrophy, and diminished mobility of the tongue, with deviation toward the affected side when protruding the tongue (9). The bilateral hypoglossal nerve palsy, however, requires the presence of specific dysarthria and dysphagia symptoms. The decreased cervical spine mobility due to cervical immobilization also caused swallowing dysfunction in this case.

Most atlanto-occipital dislocation patients who survive the injury have neurological deficits (10). C1-2 dissociation occurs with complete ligament lesions (11, 12). The identification of lesions can be difficult in cases of spontaneous reduction after subluxation (13). In this case, it is speculated that the lesion occurred at the time of the trauma. Upper cervical injuries are precarious due to a unique, complex, bony, and ligamentous vascular anatomy (14). Normal C1-2 articulations are responsible for approximately 50% of cervical spine rotation (15). This rotation narrows the space available between C1 and C2 (16). There are not many cases of isolated hypoglossal nerve palsy after injury; one case reported unilateral hypoglossal nerve palsy following a vertical atlantoaxial dislocation (17).

The reduction of C1-2 dislocation can also be achieved by a simple head extension (18) but a change in neck position at some point during transport to the hospital could have additionally contributed to the nerve compression. Halo immobilization is a less invasive treatment and can be instituted quickly and should thus be considered as one of the initial treatment options.

The previous report showed that epidural hematomas should be operated on urgently because the hematoma volume is a factor that impacts postoperative results and prognosis (19). In this case, neurological deficit due to compression by epidural hematoma of the medulla oblongata was not observed, so an intravascular embolization treatment method to treat this was selected rather than invasive surgery. In retrospect, this treatment was unnecessary as the only relevant treatment for the lesion in the case was immobilization.

Isolated bilateral hypoglossal nerve palsy secondary to trauma is an uncommon complication. Due to the extensive trauma in this case, injury to both hypoglossal nerves may have been caused by traction at the base of the skull. This bilateral hypoglossal nerve paralysis was suspected be due to atlanto-occipital dislocation rather than compression caused by the hematoma, as the progressive functional recovery and denervation activity by electromyography suggests that the pathology in the present case was caused by neuropraxic nerve damage. Anatomically, the hypoglossal nerve is located on the most lateral prominence of the anterior surface of the transverse process of the C1. Hyperextension of this joint could stretch the nerve against this prominence. It has been suggested that nerve compression and stretching against the greater horn of the hyoid bone can cause hypoglossal palsy (20, 21). Most of the cases of hypoglossal palsy reported a tendency to improve one month after the onset of symptoms and were almost completely healed by three months (22).

Magnetic resonance imaging is the best imaging technique used to diagnose an epidural hematoma (23). Unfortunately, the patient in this case could not undergo this imaging technique due to cardiorespiratory instability.

In conclusion, this case reports isolated bilateral hypoglossal nerve paralysis following an atlanto-occipital dislocation. We postulate that compression and stretching of the hypoglossal nerve against the greater horn of the hyoid bone is a probable cause of the bilateral hypoglossal palsy. If the hypoglossal palsy and surrounding symptoms did in fact result from the mechanism of compression and stretching, acute treatment and rehabilitation therapy likely supported the patient's recovery of function.

## Data availability statement

The original contributions presented in the study are included in the article/supplementary material, further inquiries can be directed to the corresponding author.

## Ethics statement

The studies involving human participants were reviewed and approved by Nara Medical University Hospital. Written informed consent to participate in this study was provided by the participants' legal guardian/next of kin. Written informed consent was obtained from the individual(s), and minor(s)' legal guardian/next of kin, for the publication of any potentially identifiable images or data included in this article.

## Patient perspective

Isolated bilateral hypoglossal nerve paralysis following an atlanto-occipital dislocation is a rare disease and appropriate immobilization therapy and rehabilitation treatment is required for a good prognosis.

## Author contributions

TM and ST: conceptualization, investigation, data curation, visualization, and writing the original draft. TM: data curation, visualization, and writing the original draft. NH: conceptualization, visualization, and writing-review and editing. AK: conceptualization, writing-review and editing, supervision, and validation. SF and NH: conceptualization and investigation. All authors took part in the final version for submission and accept overall accountability for the accuracy and integrity of the manuscript.

## Conflict of interest

The authors declare that the research was conducted in the absence of any commercial or financial relationships that could be construed as a potential conflict of interest.

## Publisher's note

All claims expressed in this article are solely those of the authors and do not necessarily represent those of their affiliated organizations, or those of the publisher, the editors and the reviewers. Any product that may be evaluated in this article, or claim that may be made by its manufacturer, is not guaranteed or endorsed by the publisher.
